# *Trichoderma asperellum* empowers tomato plants and suppresses *Fusarium oxysporum* through priming responses

**DOI:** 10.3389/fmicb.2023.1140378

**Published:** 2023-03-14

**Authors:** Amira E. Sehim, Omar A. Hewedy, Khadijah A. Altammar, Maryam S. Alhumaidi, Rasha Y. Abd Elghaffar

**Affiliations:** ^1^Botany and Microbiology Department, Faculty of Science, Benha University, Benha, Egypt; ^2^Department of Plant Agriculture, University of Guelph, Guelph, ON, Canada; ^3^Department of Genetics, Faculty of Agriculture, Menoufia University, Shebeen El-Kom, Egypt; ^4^Department of Biology, College of Science, University of Hafr Al Batin, Hafar Al Batin, Saudi Arabia

**Keywords:** *Trichoderma asperellum*, tomato, *Fusarium oxysporum*, IAA, biological control, root anatomy, confocal microscopy

## Abstract

Plant-associated microbes play crucial roles in plant health and promote growth under stress. Tomato (*Solanum lycopersicum*) is one of the strategic crops grown throughout Egypt and is a widely grown vegetable worldwide. However, plant disease severely affects tomato production. The post-harvest disease (*Fusarium* wilt disease) affects food security globally, especially in the tomato fields. Thus, an alternative effective and economical biological treatment to the disease was recently established using *Trichoderma asperellum*. However, the role of rhizosphere microbiota in the resistance of tomato plants against soil-borne *Fusarium* wilt disease (FWD) remains unclear. In the current study, a dual culture assay of *T. asperellum* against various phytopathogens (e.g., *Fusarium oxysporum*, *F. solani*, *Alternaria alternata*, *Rhizoctonia solani*, and *F. graminerarum*) was performed *in vitro*. Interestingly, *T. asperellum* exhibited the highest mycelial inhibition rate (53.24%) against *F. oxysporum*. In addition, 30% free cell filtrate of *T. asperellum* inhibited *F. oxysporum* by 59.39%. Various underlying mechanisms were studied to explore the antifungal activity against *F*. *oxysporum*, such as chitinase activity, analysis of bioactive compounds by gas chromatography–mass spectrometry (GC–MS), and assessment of fungal secondary metabolites against *F. oxysporum* mycotoxins in tomato fruits. Additionally, the plant growth-promoting traits of *T. asperellum* were studied (e.g., IAA production, Phosphate solubilization), and the impact on tomato seeds germination. Scanning electron microscopy, plant root sections, and confocal microscopy were used to show the mobility of the fungal endophyte activity to promote tomato root growth compared with untreated tomato root. *T. asperellum* enhanced the growth of tomato seeds and controlled the wilt disease caused by the phytopathogen *F. oxysporum* by enhancing the number of leaves as well as shoot and root length (cm) and fresh and dry weights (g). Furthermore, *Trichoderma* extract protects tomato fruits from post-harvest infection by *F. oxysporum*. Taking together, *T. asperellum* represents a safe and effective controlling agent against *Fusarium* infection of tomato plants.

## Introduction

Tomato (*Solanum lycopersicon* L.) is one of the most essential and widespread horticultural crops worldwide (Pritesh and Subramanian, 2011; Bawa, 2016), and it is a rich source of vitamins A and C (Pramanik and Mohapatra, 2017; Boccia et al., 2019). Egypt is a top-five tomato producer, accounting for 7.2 to 8.6 million tons annually (Nakai, 2018). However, there is a significant concern regarding food security, as demographic projections indicate that the global population will rise to 9.5 billion by 2050 (Attia et al., 2022). Food security and safety are increasingly threatened as the human population continues to expand due to soil-borne fungal pathogens emerging worldwide. In addition, such pathogens can reduce crop productivity in greenhouse and field conditions (e.g., vegetable crops), consequently causing severe losses to global food security (Almeida et al., 2019). Fungal diseases cause over 1.6 million deaths annually, and over one billion people suffer from severe fungal infections. Moreover, fungal pathogens are considered the highest threat to plant-host species, representing the main reason for reducing plant growth (approximately 65%) (Zhang and Ma, 2017; Almeida et al., 2019; Hewedy et al., 2020a,b). In addition, toxigenic fungi are the most important plant pathogens, causing diseases such as *Fusarium oxysporum* species complex (FOSC) (Zhang and Ma, 2017; Hewedy et al., 2020a,b), *Rhizoctonia solani* (Ghazala et al., 2022), *Fusarium solani* (Saengchan et al., 2022), and *Fusarium graminearum* (de Chaves et al., 2022). The plant root is the key site for interacting with the host plant, microbial pathogens, and the rhizosphere microbiome (Zhalnina et al., 2018). Furthermore, plants have become more susceptible to disease due to continuous exposure to stress and climatic changes worldwide (Attia et al., 2022). Tomato plants are affected by numerous infections caused by many different agents, including fungus, bacteria, viruses, and physiological disorders, responsible for symptoms including fruit spots, rots, wilts, and leaf spots/blights (Jones et al., 2014; Elshafie et al., 2017). *F. oxysporum* f. sp. *lycopersici* (Fol) is a potent fungal pathogen infects tomato crops by penetrating the roots. This pathogen causes yellowing of lower leaves, browning of vascular tissues, and wilting symptoms in tomato seedlings above the soil line with substantial yield losses (Borrero et al., 2012; Li et al., 2018). Moreover, infection of tomato plants is achieved by spore germination or mycelium, leading to higher plant transpiration and lower nutrient translocation, causing wilting crown and root rot and, eventually, death of the plant (Akbar et al., 2018; Manikandan et al., 2018). *Fusarium oxysporum* is a widely distributed and phylogenetically diverse species known as a mycotoxin producer (Irzykowska et al., 2012) and is ranked fifth out of the 10 most lethal (death-causing) plant pathogens. Two main formae speciales are *F. oxysporum*f. sp. *lycopersici* (FOL) and *F. oxysporum* f. sp. *radicis-lycopersici* (FORL) (Ribeiro et al., 2022). FOL is responsible for *Fusarium* wilt, and FORL causes *Fusarium* crown and root rot, among the most studied plant diseases. In Egypt, the damage to tomato crop production due to *F. oxysporum* wilt occurred in 67% of the total planted area (Selim and El-Gammal, 2015). In addition, some fungal species produce mycotoxins (e.g., aflatoxin) on plants which negatively affects the post-harvest treatments and helps to spread the causal agent between the next generation of seeds (Marshall et al., 2020). Thus, various methods, such as chemicals and pesticides, are employed to control and alleviate plant diseases (Ristaino et al., 2021). However, using fungicides to control *Fusarium* wilt is ineffective because fungal conidia stay viable for a long time, and pesticide residues are harmful to human health (Attia et al., 2022). Therefore, new control strategies must be developed with the aim of economic and environmental sustainability in plant and crop protection (Hernández-Aparicio et al., 2021). Biological control is a desirable alternative method to control the *Fusarium* wilt infection by using antagonistic nonpathogenic microorganisms to minimize the harmful effects in numerous crops (Glick, 2012). Chemical fungicides and soil fumigation have been widely used for controlling FWD disease. *Trichoderma* strains are vital anti-pathogen biocontrol agents (Pal and Gardener, 2006; Contreras-Cornejo et al., 2016; Zhang et al., 2022). This genus is a ubiquitous filamentous fungi that grow in the rhizosphere and colonizes plant roots as an opportunistic, avirulent plant symbiont (Harman, 2006). As *Trichoderma* species is a known aggressive wide range of soil fungal pathogens worldwide to suppress soil fungal diseases and plant pathogen invasion. They are frequently applied as biofungicides against pathogens such as *Botrytis cinerea*, *Fusarium* spp., *Pythium* spp., *Rhizoctonia solani*, and *Sclerotium rolfsii* on crops of economic importance (Mohiddin et al., 2010; Olowe et al., 2022; Sharma et al., 2022). *Trichoderma* fungi indirectly exert their biological control machinery toward fungal pathogens by competition for nutrients and space, antibiosis, production of growth-promoting substances (e.g., IAA, nutrient uptake) (Lei and Zhang, 2015; França et al., 2017) or by secretion of bioactive metabolites, some cell wall-degrading enzymes (CWDEs), such as chitinases and β-1,3-glucanases and mycoparasitism (Elshahawy and El-Mohamedy, 2019; Sallam et al., 2019). Mycoparasitism is the most efficient antifungal mechanism of *Trichoderma* spp. (e.g., *T. virens*, *T. harzianum*) *via* recognizing the pathogen and growing alongside the pathogen hyphae, then dissolution and death of the pathogen (Benítez et al., 2004; Sharon et al., 2007; Hewedy et al., 2020a,b; Contreras-Cornejo et al., 2021; Taylor et al., 2021; Mukherjee et al., 2022). Various factors such as native environmental habitats, strains, pathogen species, and laboratory findings for testing their antifungal activities affect the *Trichoderma* biocontrol machinery. *Trichoderma asperellum* is a promising strain based on *in vitro* assays against plant fungal phytopathogens. For example, *T. asperellum* GDFS1009 has been shown tosuppress *F. oxysporum f.* sp. *cucumerinum* (*Fusarium* wilt of cucumber) and *Fusarium graminearum* infections (Wu et al., 2017; Ketta and Hewedy, 2021), with 98 and 91% inhibition of colony radial growth, respectively, to cope with biotic stresses (Kamaruzzaman et al., 2021). In this context, the discovery of a sustainable solution is the main aim of this study to select and test a potent fungal strain to reduce the negative influence of fungal pathogenicity with the aim of controlling plant disease as well as improving plant growth and plant health to sustain food production. Thus, in this study, we sought to isolate *T. asperellum* from the rhizosphere of garlic (*Allium sativum*), identified using the ITS gene, test the antifungal activities against different fungal pathogens, and focus on the causal agent that causes FWD. Moreover, exploring the antagonistic mechanisms such as chitinase activity and bioactive secondary metabolites, as well as the role of *T. asperellum* for tomato growth promotion (e.g., IAA, P solubilization). Moreover, we present a new trend regarding post-harvest applications using fungal secondary metabolites against *F. oxysporum* mycotoxins to achieve sustainable food production in an eco-friendly environment. Finally, we used confocal microscopy as a throughput technique to explore the mobility of *Trichoderma* in the tomato roots.

## Materials and methods

### Phytopathogens and antagonistic strain

Five phytopathogenic fungi (*F. oxysporum*, *F. solani*, *F. graminearum*, *Alternaria alternata*, and *Rhizoctonia solani*) were obtained from Agriculture Research Center, Giza, Egypt. *Trichoderma* isolate (Biocontrol agent) was isolated from the garlic (*Allium sativum*) rhizosphere soil by serial dilution method. Briefly, the rhizospheric soil sample (10 g) was suspended in 90 mL of sterile distilled water, and the mixture was shaken at (200 rpm) for 30 min. Next, the soil suspension was serially diluted up to (10^−3^) dilution, then 100 μl was spread on Potato Dextrose Agar (PDA) plates supplemented with chloramphenicol (50 mg/L) and streptomycin (15 mg/L) to suppress bacterial growth (Woldeamanuale, 2017). Subsequently, the inoculated plates were incubated at 28°C for 5–7 days. At the end of the incubation period, the fungal isolate was purified, kept on slants at 4°C, and subcultured every 4 weeks (López Errasquín and Vázquez, 2003). The pure culture of the fungal isolate was characterized using morphological and microscopical characteristics (Harman and Kubicek, 2002) and confirmed by molecular identification.

### Molecular identification

Genomic DNA was extracted with the QIAamp DNeasy Plant Mini kit according to the manufacturer’s instructions. Amplification of the fungal ITS region was carried out with forward ITS1 (5′ TCC-GTA-GGT-GAA-CCT-GCG-G 3′) and reverse ITS4 (5′TCC-TCC-GCT-TAT-TGA-TAT-GC 3′) primers (White et al., 1990). The polymerase chain reaction program was performed as previously described (Hewedy et al., 2020c). The homology of the ITS rDNA sequence of the isolate was analyzed using the BLAST program from the GenBank database.^1^

### *In vitro* antagonistic activity of *Trichoderma asperellum* against phytopathogenic fungi

The antagonistic activity of *T. asperellum* against fungal pathogens was evaluated by dual culture assay (Awad et al., 2018). One mycelial disk of the pathogen (6 mm diameter) was deposited on one side of the Petri plate, and another disk of (antagonist) was deposited equidistantly on the other side. PDA plates containing only disks from the pathogen mycelium were used as controls. All the experiments were conducted in triplicates. After 7 days of incubation at 28°C, the radial growth of the phytopathogens in the control and treatment plates was measured, and the percentage of inhibition of radial mycelial growth (PIRG) was calculated using the following equation:


%PIRG=R1−R2/R1×100

where *R*1 = radial growth of the phytopathogen in the control plate; *R*2 = radial growth in the presence of an antagonist (Fishal et al., 2022).

### Effect of *Trichoderma asperellum* culture filtrates on *Fusarium oxysporum*

Based on the dual culture assay results, the maximum growth inhibition was recorded against *F. oxysporum*. Thus, different concentrations of the culture of *T. asperellum* filtrate were tested against *F. oxysporum*. *T. asperellum* was inoculated in 50 mL of potato dextrose broth and incubated at 28°C for 14 days on a rotary shaker at 150 rpm. Cell-free supernatant was obtained by filtration through Whatman filter paper No. 1. The filtrate was sterilized using a 0.2 μm pore biological membrane filter (Jangir et al., 2019). Each sterilized filtrate was mixed into a PDA medium to obtain 5, 15, and 30% (v/v). Finally, 20 mL of the medium was amended with different filtrate concentrations and poured into 90 mm Petri plates. The secondary metabolites in the filtrate were tested for their efficacy against the test pathogens. The test pathogen was centrally inoculated with an individual equal disk (5 mm) of seven-day-old culture. PDA plates inoculated with pathogens without culture filtrates served as a control. Three replicates were maintained for each treatment and incubated at 28°C. The percentage inhibition of mycelial growth was calculated as mentioned above.

### Scanning electron microscopy

The confrontation of hyphal interaction between *F. oxysporum* and *T. asperellum* was also examined by SEM. Briefly, a mycelial disk (5 mm) of both microbes was cultured on PDA for 4–5 days of incubation. The plate cultures were examined under a light microscope to check the early touch stage. The contact interactions were labeled, and 1 cm agar blocks were removed for SEM preparation, fixed with osmium tetroxide, and then dehydrated using a serial dilution of the ethyl alcohol and acetone. A critical point drier (Tousimis Autosamdri–815 Coater) was then used to dry the processed samples with gold using a sputter coater. Then, the mycoparsatism and the hyphae interactions were examined by SEM (JEOL JSM 6510 lv). The microscope was operated at 30 KV at EM Unit, Mansoura University, Egypt. Three replicates were included for each sample (Su et al., 2010).

### Assessment of plant growth-promoting and antifungal properties of *Trichoderma asperellum*

#### Indole-3- acetic acid

*Trichoderma asperellum* was grown on potato dextrose broth (PDB) supplemented with L-Tryptophan (0.1 g/L) for 4–5 days at 28 ± 2°C on a rotary shaker at 150 rpm (Nandini et al., 2021). Next, both mycelium and debris were separated by filtration and centrifugation, respectively. Briefly, 1 mL of the culture filtrate and 4 mL of Salkowski reagent (1 mL of 0.5 M ferric chloride in 50 mL of 35% perchloric acid) were mixed and kept for 30 min in the dark for color change. The generation of pink indicated the production of IAA. The optical density was detected and measured at 530 nm using a spectrophotometer (JENWAY 6315). A standard curve of IAA concentrations was designed to evaluate the corresponding concentration of IAA-released *T. asperellum* in the bioassay media.

#### Phosphate solubilization efficacy of *Trichoderma asperellum*

A plug of *T.asperellum* (5 mm) was grown on a modified NBRIP medium (Nautiyal, 1999) supplemented by Bromophenol blue as an indicator for 7 days at 28°C. The medium contained the following composition (gL^−1^: 10.0 glucose), 0.5MgCL_2_.6H_2_O, 0.25MgSO_4_.7H_2_O, 0.2KCL, 0.1 (NH_4_)_2_SO_4_ and 15.0 agar, with the addition of 50 mL of K_2_HPO_4_ (10% w/v) and 100 mL of CaCL_2_ (10% w/v) to precipitate insoluble calcium phosphate (CaHPO_4_). The pH of the medium was adjusted to 7.0. After incubation, a yellow halo zone around the fungal colony indicated phosphate solubilizing ability. The solubilization zone and colony diameters were measured and calculated using the equation below to indicate the solubilization efficiency (Pande et al., 2017; Aloisio et al., 2019).

SE = (Solubilization diameter)/(Growth diameter) ×100.

#### Chitinase activity of *Trichoderma asperellum*

Chitinase detection medium with ingredients/per liter: 0.3 g of MgSO_4_.7H_2_O, 3.0 g (NH4)_2_SO_4_, 2.0 g of KH_2_PO_4_, 1.0 g of citric acid monohydrate, 15 g of agar, 200 μl of Tween-80, 4.5 g of colloidal chitin and 0.15 g of bromocresol purple pH (4.7), was prepared and autoclaved at 121°C for 15 min. Then, the plates were inoculated with a fresh culture plug of *T. asperellum*, incubated at 25 ± 2°C for 7 days, and were observed for colored zone formation (Agrawal and Kotasthane, 2012).

### *Trichoderma asperellum* promotes tomato plant growth

#### Inoculum preparation

Seven-day-old pure culture of *T. asperellum* was used. After cultivation on PDA slants and incubation at 28°C, sterile water was poured into the slants, and the spores were scraped with a sterile glass rod. The spore suspension was filtered and put into tubes, and the spore concentration of *T. asperellum* was adjusted at 1 × 10^6^ spore/mL (Scudeletti et al., 2021; Natsiopoulos et al., 2022).

#### Effect of *Trichoderma asperellum* on tomato seeds germination and growth promotion

Tomato seeds of (Hybrid Madera F1) were used in all experiments. The tomato seeds were surface sterilized with 2.5% (v/v) sodium hypochlorite for 10 min and rinsed three times with distilled water before seed inoculation. Afterward, the seeds were immersed for 24 h in fungal suspensions prepared at 1.0 ×10^6^ spore mL^−1^. The control treatment consisted of seeds immersed only in sterile distilled water for the same period. The seeds were deposited in Petri dishes containing autoclaved tissue papers moistened with sterile water (16 seeds per dish) that were sealed with parafilm to prevent evaporation and then incubated at 28°C for 7 days (Kipngeno et al., 2015; Barroso et al., 2019). Each treatment was replicated three times. The germination rate (%) was estimated by counting the number of seeds germinating after one week of cultivation.

Three sterilized tomato seeds were transferred to one side of the plate prepared with water agar containing 0.2% (w/v) glucose (Yoo et al., 2018). The other side contained a *T. asperellum* disk (5 mm) grown on PDA. Plates without inoculum were considered a control, and the plates were incubated for 3–4 days in darkness at 28°C. After the incubation period, the germination percentage (GP) was evaluated according to the following equation:


GP(%)=n/N×100

where: *n* = number of germinated seeds; *N* = total number of seeds.

Next, treated and untreated (control) seeds were sown in pots (8 cm) in diameter containing a mixture of sterilized peat moss and soil (1:2 w/w) at 28 g/pot, with a density of five seeds per pot. Pots were maintained under controlled conditions in the growth chamber (22 ± 3°C). Experiments were performed using a completely randomized design (CRD) with two treatments and three replicates. The pots were rotated three times a week to ensure uniform growth conditions in the growth chamber. After 21 days, plant samples were taken to measure the shoot height (cm), the root length (cm), and plant biomass. The seedling vigor index (VI) was calculated as described by Buriro et al. (2011).


Seedling vigor index=[seedling length(cm)×GP(%)]

#### Confocal microscopic assay

The interaction between *T. asperellum* strain and the tomato-root system during the early stages of root colonization was examined by confocal microscopy. SYTOX Green (Thermo Fisher Scientific-United States) was used for *Trichoderma* staining. Briefly, tomato roots were carefully removed from the sterilized soil and gently swirled 4–5 times in autoclaved water to wash the clay particles. Then, the primary root was stained using 10 μM propidium iodide (PI) for 10 min to label the plant cell wall and washed by ddH_2_O 4–5 times. Subsequently, the root was placed on a glass slide to facilitate the visualization of the localization of *T. asperellum* (green) in the tomato root (red) surface as a plant growth promoter (Chacón et al., 2007).

#### Estimation of the biocontrol efficiency of *Trichoderma asperellum* against *Fusarium* wilt disease

Sterilized tomato seeds were sown in pots (8 cm) in diameter containing 28 g of sterilized peat moss and soil (1:2, w/w). Each pot contains five seeds, and the experiment included four treatments as follows:

1. Control (distilled water) only.
2. Pathogen (*F. oxysporum*) only.
3. Bioagent (*T. asperellum*) only.
4. *Trichoderma* + *Fusarium.*

Tomato plants were inoculated and kept at 25°C for 21 days, and the parameters of plant phenotype were measured, including the root length, shoot, and fresh weight. The shoots and the roots were dried in an oven at 70°C until constant weight (Mwangi et al., 2011).

#### Tomato root anatomy under biotic stress

The roots of tomato plants were sectioned and studied anatomically. The wax method was used in this experiment (Johansen, 1940). Briefly, plant roots were fixed for 72 h using FAA (formalin: acetic acid: alcohol 90:5:5) fixative. Then, roots were washed several times using distilled water and dehydrated using serial concentrations of alcohol 50, 70, 90, 95, and 100% (v/v), respectively. For clearing, they were transferred every 3 h from a mixture of 1:1 cedarwood oil: and absolute alcohol into pure cedarwood oil, followed by a mixture of cedarwood oil and xylene, and left overnight in pure xylene. Wax embedding was carried out in an oven adjusted at 60°C, embedded in clear wax, and sectioned using a rotatory microtome. Staining was done using safranine and fast green stains. Sections were mounted in a drop of Canada balsam, covered, and left to dry. The prepared slides of each root treatment were repeatedly examined under the light microscope (LEICA DM750) at the Microscopy Department, Faculty of Agriculture, Cairo University.

### *Trichoderma asperellum* protects tomato fruits against FWD

#### Preparation of *Trichoderma asperellum* crude extract

Bioactive secondary metabolites of *T. asperellum* were extracted using ethyl acetate solvent, as previously described by Chen et al. (2018). *Trichoderma* was grown in a PDB medium and incubated on a rotary shaker (150 rpm) for 21 days at 28°C. After the incubation period, the fermented broth was filtered through Whatman filter paper No. 1, and the metabolites produced by the fungus were extracted from an equal volume of ethyl acetate. An equal volume of ethyl acetate was added to the filtrate and vigorously shaken for 5 min at room temperature. The mixtures were transferred to separating funnels, and the organic layers of ethyl acetate were allowed to separate from the aqueous layers. Then, the ethyl acetate layer was allowed to dry at room temperature, and the dried extracts were stored at 4°C for further use.

#### Assessment of extracted secondary metabolites against mycotoxigenic *Fusarium oxysporum* in tomato fruits

The antifungal activity of the *T. asperellum* extract was evaluated against *Fusarium* on tomato fruits, which were obtained from the supermarket, were initially washed under running water, sterilized for 2 min in 2% (v/v) sodium hypochlorite, and then rinsed with sterile water. Watery fruits were placed in plastic trays (sterilized with 70% (v/v) ethanol and under UV) and dried for 2 h under a laminar flow cabinet. The fruits were wounded with a sterile needle, and 20 μl of the pathogen spore suspension at 10^4^ spores/mL was inoculated onto the wound. Subsequently, the drop was dried for 1 h. Finally, tomato fruits were treated with 20 μl of *Trichoderma* crude extract in the same wound where the pathogen inoculum had been applied. The treated tomato fruits were dried under a laminar flow cabinet until the droplet was completely dry, and the closed trays were incubated at room temperature (Stracquadanio et al., 2021; Maliehe et al., 2022). The untreated positive control was inoculated with the spore suspension of the pathogen, and the negative control was inoculated with *Trichoderma* extract. Fruits were monitored daily, and results were evaluated on the 11th day of incubation. The diameter of the lesions was also observed.

#### Mycotoxin analysis

Two grams of ground tomato fruits were mixed with 8 mL (80 acetonitrile (ACN): 20 H_2_O) and shaken for 20 min. Then, the mixture was centrifuged at 3500 rpm for 10 min and filtered through a 0.45 μm nylon filter. After filtration, the extract was diluted (1:4) with water containing 5 mM ammonium acetate. Analysis of mycotoxins (FB1, FB2, DON, and ZEN) was performed using liquid chromatography-electrospray ionization–tandem mass spectrometry (LC-ESI-MS/MS) with an ExionLC AC system for separation and SCIEX Triple Quad 5,500+ MS/MS system equipped with electrospray ionization (ESI) for detection. The instrument data were collected and processed using the SCIEX OS 1.6.10.40973 software. The targeted analytes were separated with an Agilent Zorbax Eclipse Plus C18 Column (4.6 × 100 mm, 1.8 μm) (Waskiewicz et al., 2010). The mobile phases consisted of two eluents containing 10 mM ammonium formate, eluent A was 0.1% (v/v) formic acid in the water, and eluent B was 0.1% (v/v) formic acid in methanol (LC grade). The mobile phase gradient was programmed as follows: 10% B at 0 min, 10–30% B from 0.0–2.0 min, 30–100% B from 2.0–11.0 min, 100% B from 11.0–11.5 min, 100–10% B from 11.5–12.0 min, 10% B from 12.0 to 15.0 min. The flow rate was 0.6 mL/min, and the injection volume was 10 μl. For MS/MS analysis, positive ionization mode (+MRM) was applied with the following parameters: curtain gas: 20 psi; collision gas: 9 psi; nebulizer current: 3; source temperature: 600°C; ion source gas 1 (nebulizer gas): 60 psi.

#### GC–MS analysis

The chemical composition of *T. asperellum* crude extract was assessed to detect the active constituents exhibiting antifungal activity. GC–MS was performed using a Trace GC1310-ISQ mass spectrometer (Thermo Scientific, Austin, TX, United States) with a direct capillary column TG–5MS (30 m × 0.25 mm × 0.25 μm film thickness). The column oven temperature was initially held at 50°C and then increased by 5°C/min to 230°C for 2 min, then increased to the final temperature of 290°C by 30°C /min and held for 2 min. The injector and MS, transfer line temperatures, were kept at 250°C and 260°C; Helium was used as a carrier gas at a 1 mL/min constant flow rate. The solvent delay was 3 min, and diluted samples of 1 μl were injected automatically using Autosampler AS1300 coupled with GC in the split mode. In full scan mode, EI/MS were collected at 70 eV ionization voltages over m/z 40–1,000. The ion source temperature was set at 200°C, and the components were identified by comparing their retention times and mass spectra with those of the WILEY 09 and NIST 11 mass spectral databases (Mulatu et al., 2022).

#### Statistical analysis

Analysis of variance (ANOVA) was assessed using IBM SPSS Statistics Version 28. The experiments were conducted using a completely randomized design with three replicates. The growth of the fungal strains was compared by t-test, and their biocontrol activities were evaluated by Fisher’s least significant difference (LSD) test at the 5% significance level.

## Results

### Isolation, morphological and molecular identification

**Figure 1:**
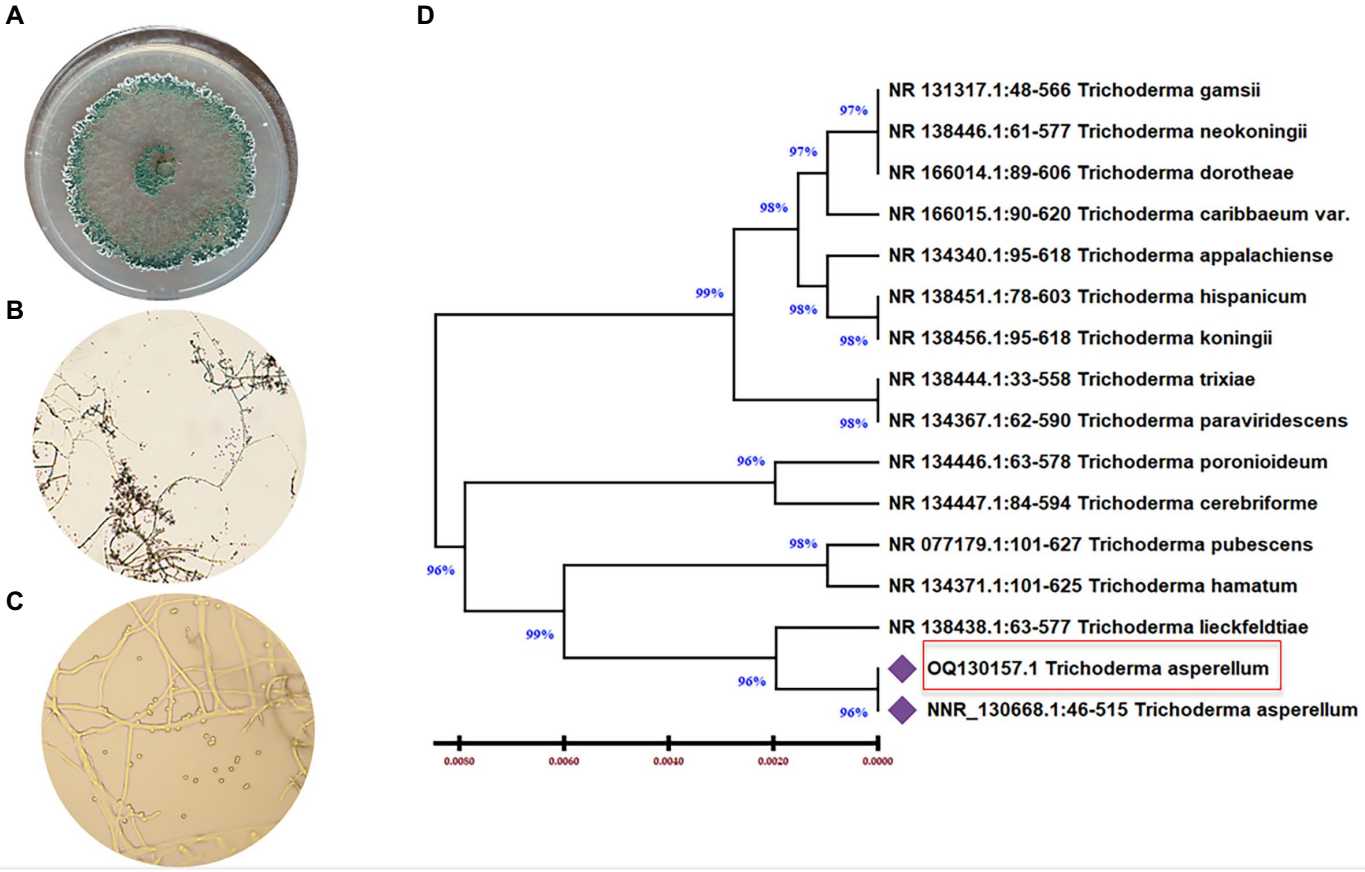
Morphological and molecular identification of *T. asperellum*. **(A)** The cultural view of T*. asperellum* grown on synthetic nutrient agar (SNA) media at 28°C for five days, which shows a ring around the original inoculum, **(B,C)** Microscopic view of conidia and conidiophores. **(D)** Molecular phylogenetic based on rDNA internal transcribed spacers (ITS) analyzes of *T. asperellum* by maximum Likelihood Model of MEGA11.0. The evolutionary distances were computed using the Maximum Composite Likelihood method and are in the units of the number of base substitutions per site. The proportion of sites where at least 1 unambiguous base is present in at least 1 sequence for each descendent clade is shown next to each internal node in the tree. This analysis involved 16 nucleotide sequences. All ambiguous positions were removed for each sequence pair (pairwise deletion option). The percentage of replicate trees in which the associated taxa clustered together in the bootstrap test (500 replicates) are shown next to the branches. The tree is drawn to scale, with branch lengths in the same units as those of the evolutionary distances used to infer the phylogenetic tree.

*Trichoderma asperellum* was isolated from garlic rhizosphere soil by plate dilution, and the pure culture was maintained on a Potato dextrose agar (PDA) medium. Macroscopic morphology of *T. asperellum* revealed the rapid growth of the colony (3–4) days with 1–2 concentric rings. The mycelium, initially of a white color, acquired green and yellow shades or remained white due to the abundant production of conidia (Figure 1A). Regarding microscopic observations, *T. asperellum* showed globous, subglobous, or ovoid conidia, ampuliform phialides, and branched conidiophores, as shown in Figures 1B,C. The identification of the fungal isolate was further confirmed by analysis of the ITS sequence. One band of 531 bp was amplified using ITS primers, and the sequence alignments were performed using the Basic Local Alignment Search Tool (BLAST) to determine the phylogenetic positions of our strain with other *Trichoderma* strains on the GenBank. The fungal strain had (100%) matched identify with *T. asperellum* (Figure 1D). Moreover, the ITS sequence of *T. asperellum* was submitted to GenBank under the accession number (OQ130157).

### Evaluation of the antagonistic activity of *Trichoderma asperellum* against phytopathogens

**Figure 2:**
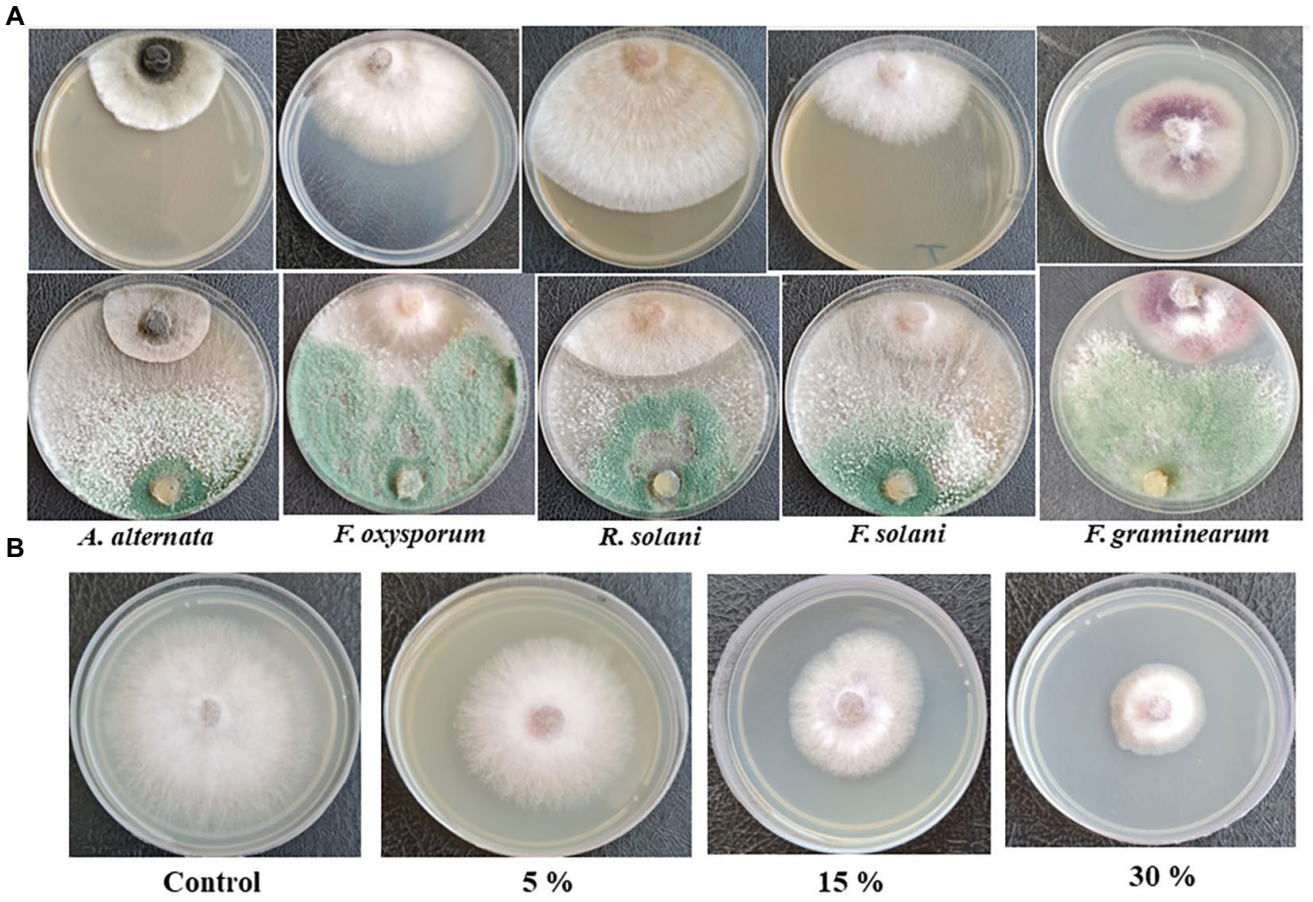
**(A)** Antagonistic activity of *T. asperellum* against plant pathogenic fungi in dual culture assay, **(B)** Effect of different concentrations of *T. asperellum* filtrate on *F. oxysporum* growth.

*Trichoderma asperellum* was tested against several plant pathogenic fungi (*F. oxysporum*, *F. solani*, *F. graminearum*, *A. alternata*, and *R. solani*) to assess the antifungal activity using dual culture assay. Results showed that *T. asperellum* could inhibit the radial mycelial growth of all tested pathogens. The maximum growth rate inhibition (53.23%) was recorded against *F. oxysporum* followed by *F. graminearum* (45%), *F. solani* (43.19%), and *R. solani* (30.89%). In contrast, the lowest inhibitory activity percentage was exhibited against *A. alternata* (20.67%) (Table 1; Figure 2A). Based on the dual culture assay results, the highest growth rate inhibition was displayed against *F. oxysporum*. Therefore, different concentrations of sterilized culture filtrate of *T. asperellum* were tested against *F. oxysporum* to evaluate the optimum inhibiting concentration. Results showed that the inhibitory activity increased by increasing the sterilized culture filtrate concentration, and the maximum inhibitory activity percentage (59.38%) was observed at a concentration of 30% (Table 2; Figure 2B).

**Table 1:** Antagonistic activity of *T. asperellum* against some phytopathogenic fungi.

Bioagent	Phytopathogenic fungi									
	*F. oxysporum*		*F. solani*		*F. graminearum*		*A. alternata*		*R. solani*	
	RMG (cm)	PIRG (%)	RMG (cm)	PIRG (%)	RMG (cm)	PIRG (%)	RMG (cm)	PIRG (%)	RMG (cm)	PIRG (%)
*Control*	6.43 ± 0.03	0.00	5.63 ± 0.15	0.00	6.00 ± 0.05	0.00	4.16 ± 0.03	0.00	7.18 ± 0.11	0.00
*T. asperellum*	3.01 ± 0.04	53.23	3.20 ± 0.17	43.19	3.30 ± 0.11	45	3.30 ± 0.05	20.67	4.96 ± 0.12	30.89

RMG, Radial Mycelial growth; PIRG, Percent inhibition of Radial Mycelial growth; ± SE.

**Table 2:** Effect of different concentrations of *T. asperellum* culture filtrate against *F. oxysporum.*

Culture filtrate concentrations (%)	*F. oxysporum* (RMG) (cm)	PIRG (%)
Control	7.63 ± 0.03	0.0
5	5.34 ± 0.08	30
15	4.65 ± 0.02	39.03
30	3.10 ± 0.05	59.38

RMG, Radial Mycelial growth; PIRG, Percent inhibition of Radial Mycelial growth; ± SE.

### Scanning electron microscopy

**Figure 3:**
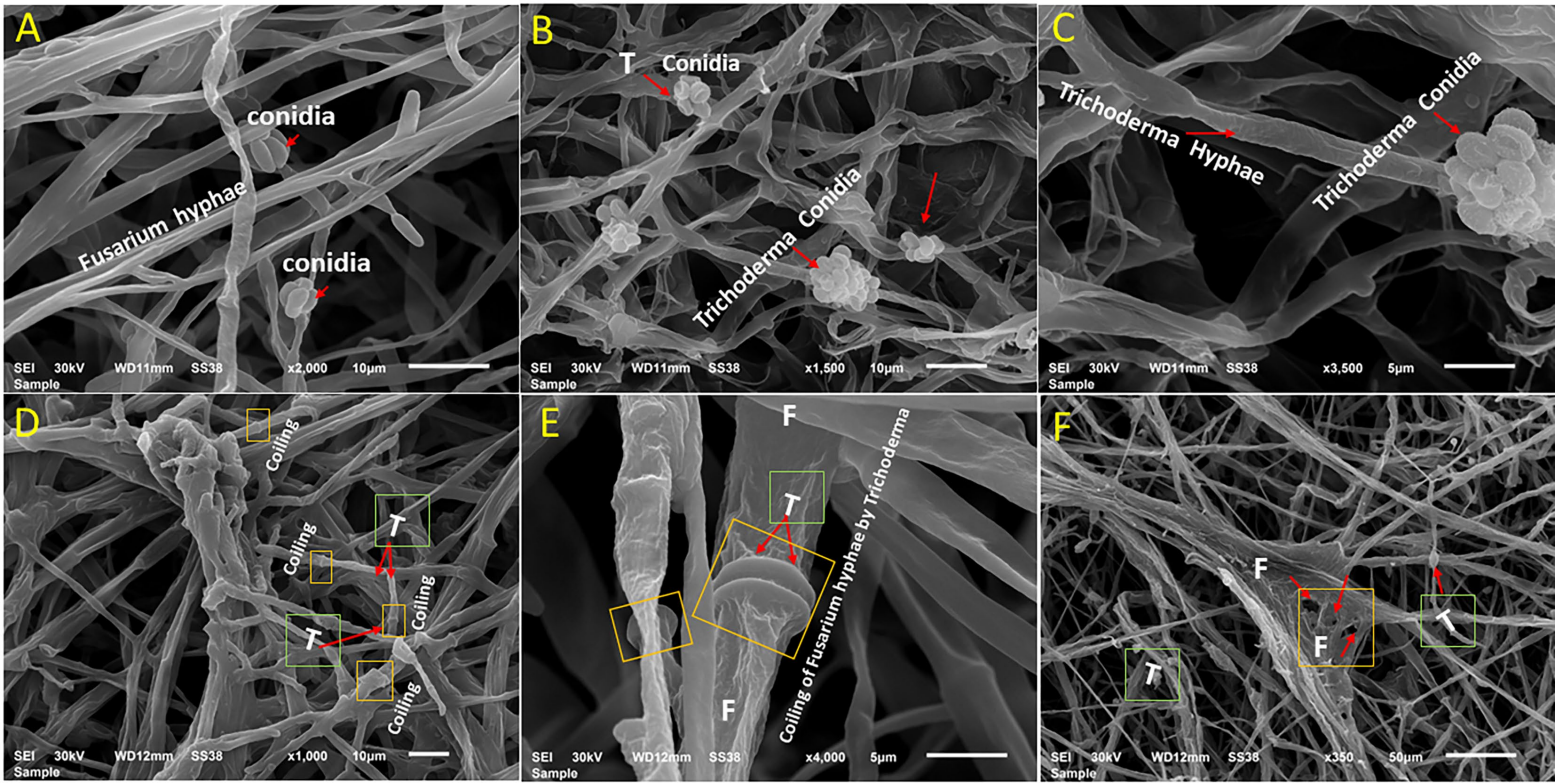
Scanning electron microscopy interpretations of the mycoparasitic of *T. asperellum* on *F. oxysporum*. **(A)** *Fusarium* pathogen alone, **(B,C)** *T. asperellum* alone (endophytic hyphae and conidia), **(D)** Mycelium of both fungi in contact *via* growing of *Trichoderma* hyphae (T) over *Fusarium* hyphae, **(E)** Coiling of *Fusarium* hyphae (F) by *Trichoderma* (T) as one of the antagonistic mechanisms (arrows), **(F)** Deformation of *F. oxysporum* by *T. asperellum.*

The mycoparasitic nature of *T. asperellum* on *F. oxysporum* as a dual culture was examined by SEM (Figure 3). Interestingly, *T. asperellum* hyphae grew over the hyphae of *F. oxysporum*, followed by quick and excessive coiling. Finally, *F. oxysporum* lysis was eventually observed (Figures 3D–F).

### Plant growth-promoting and antifungal properties of *Trichoderma asperellum*

**Figure 4:**
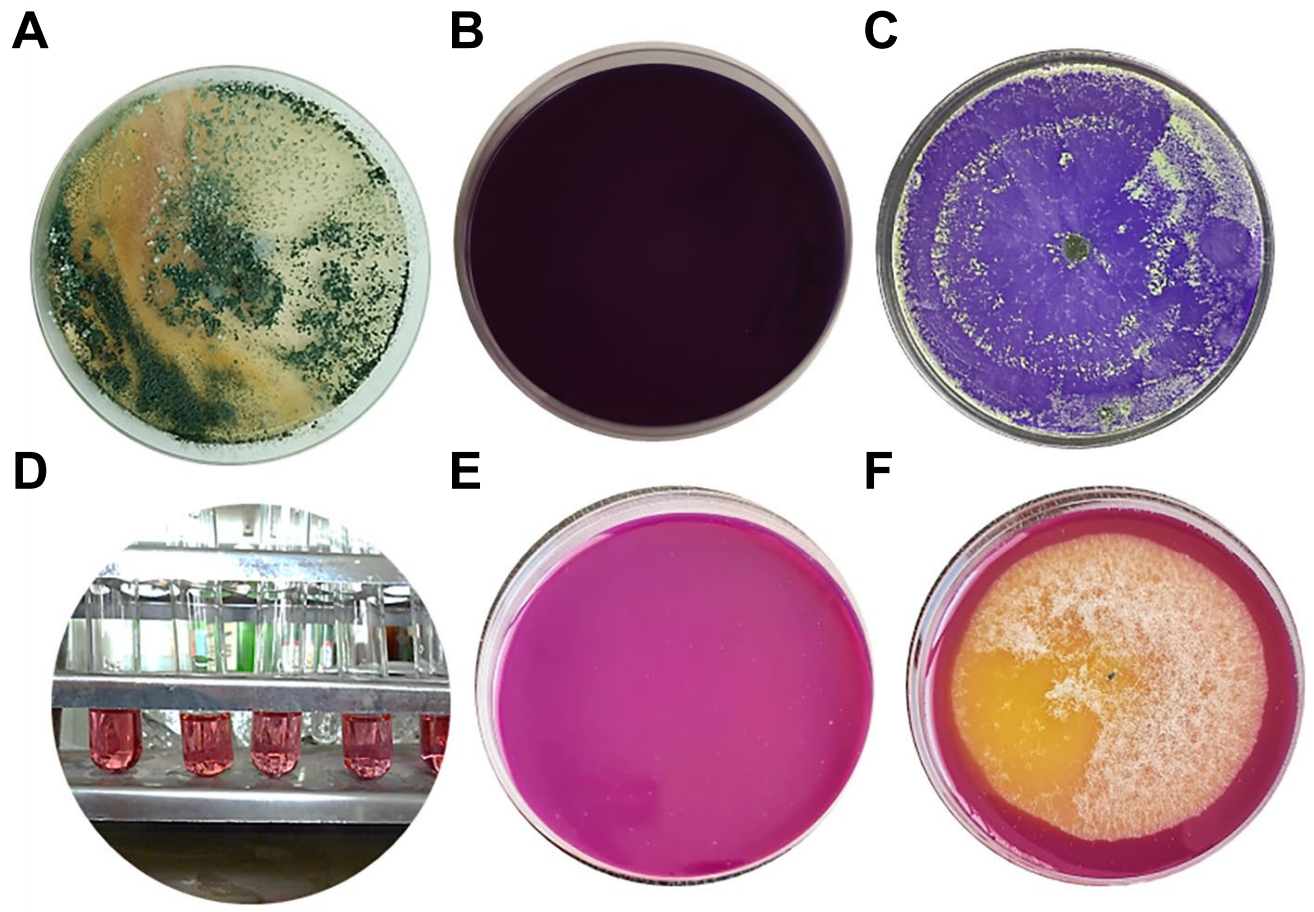
**(A)** *Trichoderma asperellum* strain grown on PDA at 28°C for seven days. **(B,C)** Screening of *T. asperellum* for chitinase activity on medium supplemented with colloidal chitin and control without inoculum. **(D)** IAA production by *T. asperellum* (5 replicates). **(E,F)** Phosphate solubilization of *T. asperellum* on NBRIP media supplemented with bromophenol blue as an indicator. **(E)** Uninoculated (control), **(F)** inoculated with *T. asperellum.*

To assess the growth-promoting effects of *T. asperellum* on plant development, the plant growth hormone IAA was measured using a colorimetric assay. Results showed that the amount of IAA produced by *T. asperellum* was (12.5 ± 0.5742μgmL^−1^). Moreover, *T. asperellum* could solubilize phosphate, exhibiting phosphorus solubilization indices of 100% (Figures 4E,F). Additionally, *T. asperellum* showed high chitinase activity in a colloidal chitin medium supplemented with bromocresol purple (with final acidic pH of 4.7). *Trichoderma*, breakdown chitin to N- acetyl glucosamine shifting pH toward the alkalinity, which changes the medium color to purple (Figures 4B,C).

### Effect of *Trichoderma asperellum* on tomato seeds germination and growth promotion

**Figure 5:**
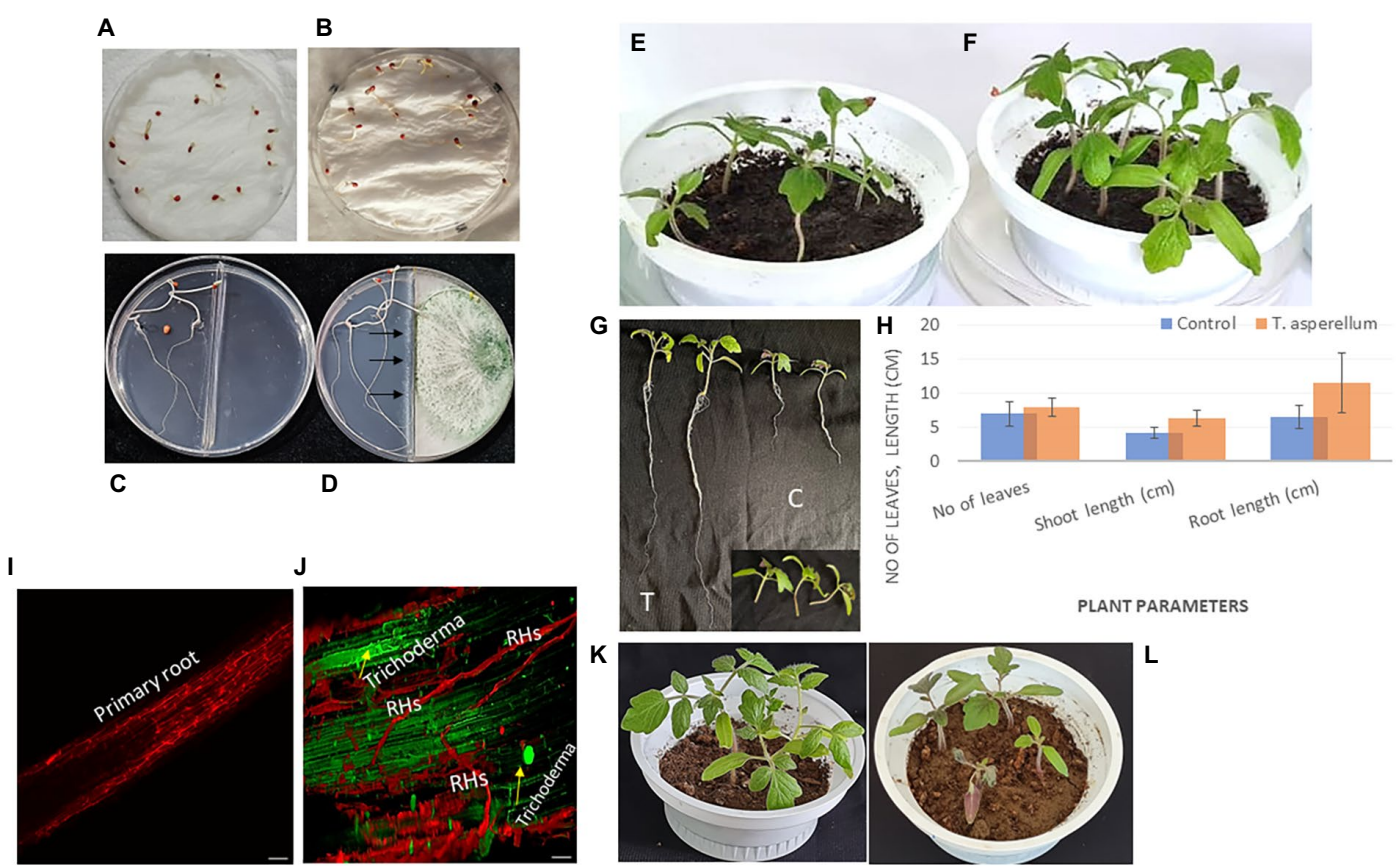
*Trichoderma asperellum* colonizes a tomato plant’s root system and promotes root elongation. **(A)** Seeds treated with distilled water. **(B)** Seeds treated with a spore suspension of *T. asperellum*. **(C)** Split interaction of seeds growing on water agar medium without *Trichoderma* treatment (control). **(D)** Split interaction with *Trichoderma* treatment. **(E,F)** Tomato plants are grown in the presence of *Trichoderma* after seed coating (pot at right) and without microbial inoculation (control only) (pot at left). **(G)** Promotion of root and shoot length *via Trichoderma* inoculation (T) compared to untreated plants (C). **(H)** Plant parameters include the number of leaves, shoot length, and root length with *Trichoderma* inoculation and control treatment. *In vivo* tests indicated the growth-promoting potential of this fungal endophyte. *Trichoderma* could boost plant growth by activating either an individual or numerous mechanisms. **(I,J)** Representative confocal laser scanning microscopical analysis illustrating the early and robust colonization of tomato roots/root hairs (RHs) by *T. asperellum* compared with the control (without microbial inoculation) using CLSM with a Leica TCS SP2. **(K,L)** Tomato plants are grown in the presence of *T. asperellum* after three weeks from seed coating (pot at left) and plants infected with *Fusarium* (pot at right).

Data illustrated in Figure 5; Supplementary Figure S1 indicated that *T. asperellum* treatment enhanced seed germination, root and shoot length, and vigor index in tomato seedlings. After 7 days, seeds treated with spore suspension of *T. asperellum* (10^6^ spore mL^−1^) resulted in the highest seed germination rates (100%), as compared to the control (81.25%). Also, it can be seen that in the presence of *T. asperellum* grown on PDA in split interaction, the germination rate of seeds was enhanced (100%) as compared to an un-inoculated plate (66.6%) as presented in Figures 5C,D. In addition, *T. asperellum* inoculation promoted tomato growth parameters (Figures 5E,F). We observed that the Average shoot length of plants inoculated with *T. asperellum* simultaneously was 14.5% longer than the control. The average root length was also 17.6% longer than the control treatment (Figures 5G,H).

### Confocal microscopy

The capacity of the fungus *T. asperellum* to colonize tomato roots and stimulate plant growth was observed using a confocal imaging assay. Sterilized tomato seeds were inoculated with *Trichoderma* spores before germination on Petri dishes (150 mm) and kept in the dark for 3 days to study the early stages of the fungal root colonization. Interestingly, the root elongation of tomato seedlings and intercellular hyphal growth were observed compared with the uninoculated treatment (control) (Figures 5I,J). To obtain more insight, an *in vitro* experiment was carried out to study the effect of a biocontrol agent at the initial stage of root colonization by *T. asperellum* to understand its influence on tomato growth promotion. After inoculation, tomato roots were covered with conidia. Initial contact between conidia and the root was primarily at the region of root hairs.

### Effect of *Trichoderma asperellum* on the growth of tomato plants under biotic stress of *Fusarium oxysporum*

**Figure 6:**
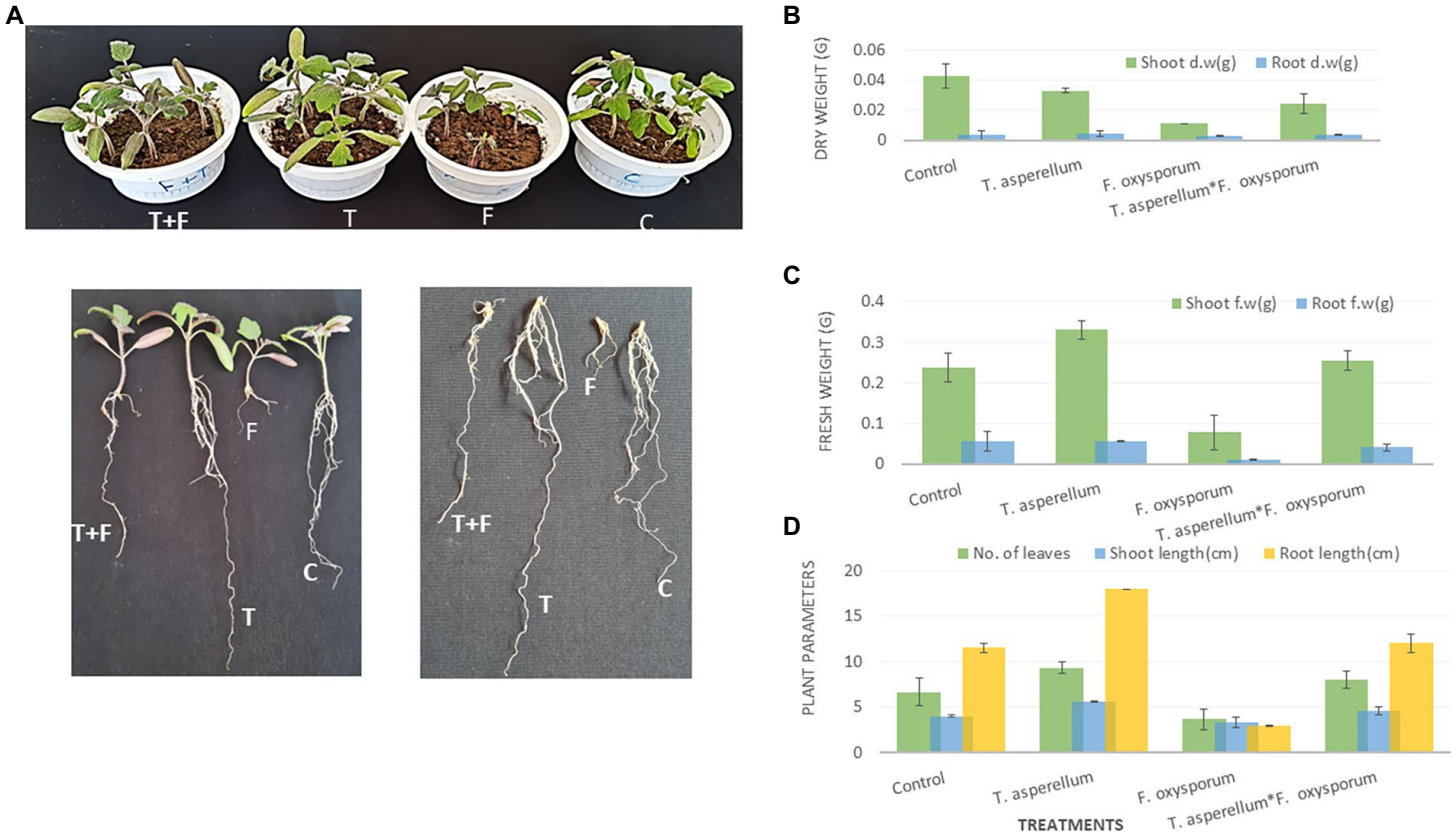
The Effect of *T. asperellum* on the growth of tomato plants under biotic stress of *F. oxysporum* including **(A)**; T-*Trichoderma* enhances both root and shoot of tomato seedlings, F-*Fusarium* can penetrate tomato roots and cause wilting or root rotting diseases, T + F-the interaction between the fungal endophyte and the pathogen, C-tomato plants without microbial treatment. Shoot and root dry weight **(B)**. Shoot and root fresh weight and **(C)**. The number of leaves, shoot, and root length (cm) **(D)**.

There was a notable variation between the tomato plants inoculated with *Trichoderma* and *Fusarium* individually, as shown in Figure 6A. Data presented in Figures 5K,L showed that *T. asperellum* improved the growth of tomato plants under *Fusarium* infection. In addition, Figures 6B–D showed a significant increase in the root length and the fresh weights of shoots between the control (untreated) and the inoculated tomato plants with *Trichoderma* under biotic stress. The inhibitory effects of *Trichoderma* inoculation against *Fusarium* invasion were demonstrated in Figure 6A. *Fusarium* pathogen invaded the root system and significantly decreased roots and shoot dry weights, the number of leaves, and root length. In addition, there was a marked increase in the root length and shoot fresh weight during the *Trichoderma-Fusarium* interactions (Figures 6C–D). *T. asperellum* controlled the FWD caused by *F. oxysporum* by enhancing the plant phenotypes, including shoot and root length (cm), shoot and fresh root weight (g), shoot and root dry weight (g), as well as the number of leaves. It can be seen that number of leaves in plants inoculated with *Trichoderma* was (45%) higher than the number of leaves in the treated plants with *Fusarium* alone.

### Root anatomy

**Figure 7:**
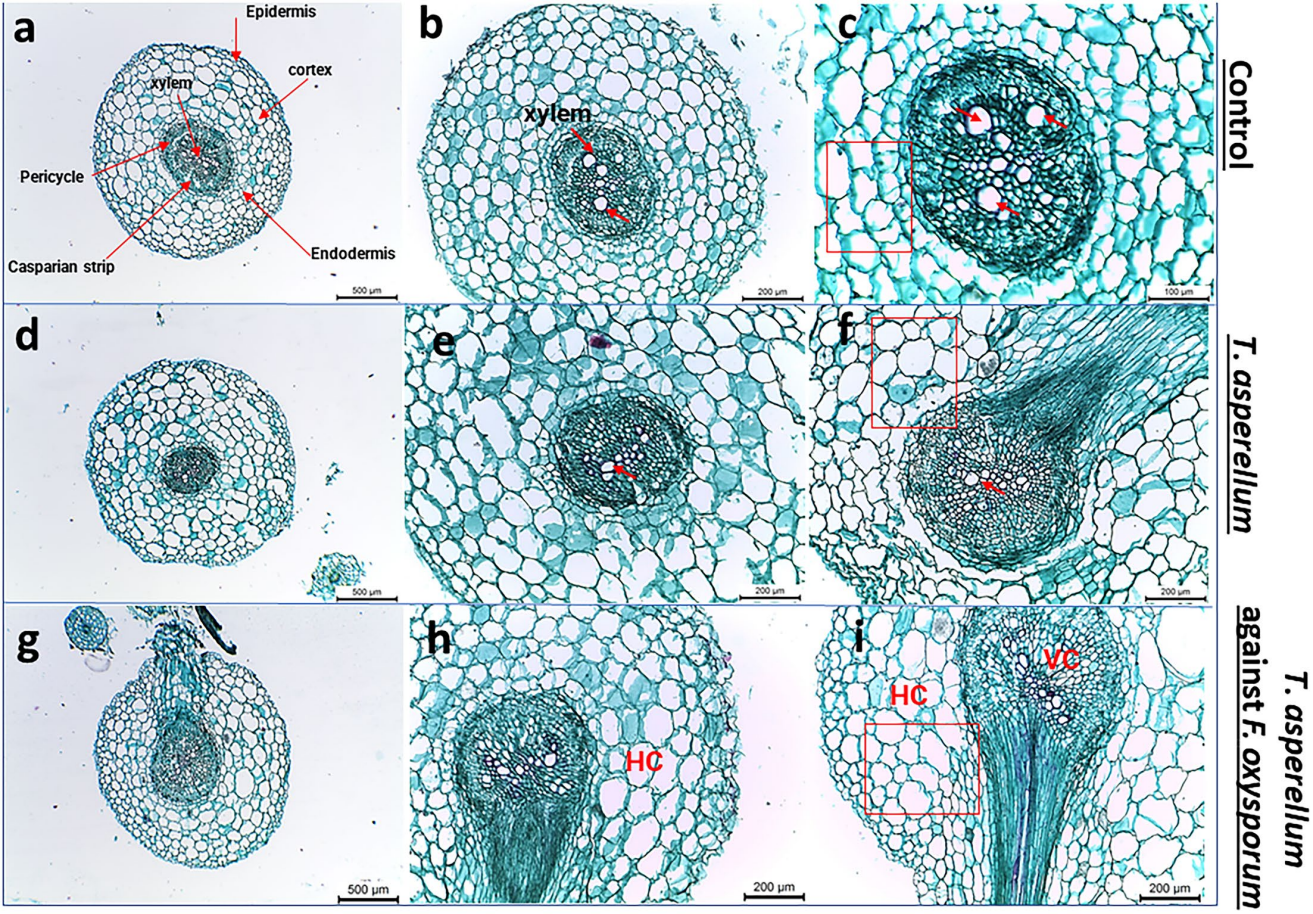
Light microscope images of tomato root cross sections near the root tip taken from plants grown under biotic stress. **(A–C)** Healthy tomato root, **(D–F)** Transverse sections of tomato roots plant treated with *Trichoderma*, **(G–I)** shows the root cross-sections after applying *Trichoderma* against *Fusarium* pathogen. Red arrows indicate the xylem vessels.

Transverse sections of tomato roots treated with *T. asperellum* and the interactions between bioagent and the fungal pathogen were studied to evaluate their impact on root health. Interestingly, the inoculation of tomato roots with *Trichoderma* increased whole root thickness and the number of xylem vessels compared to the control (Figures 7A,D,G). The highest degradation area of the root cortex was observed in plants infested with *F. oxysporum*. Lateral root (LR) development was also detected in the treated tomato roots with *T. asperellum* (Figure 7F). However, the combination of *T. asperellum* and *F. oxysporum* enhanced the root thickness in the cortex of the tomato tap root (Figure 7I). Light microscope sections showed that *Trichoderma* enhanced the cortex and xylem diameters. It was also noted that the xylem and the endodermis diameters were thinner in control roots than in other treatments; this trend was the same in both hybrids (Figures 7C,F,I).

### Biocontrol efficiency on tomato fruits against FWD

**Figure 8:**
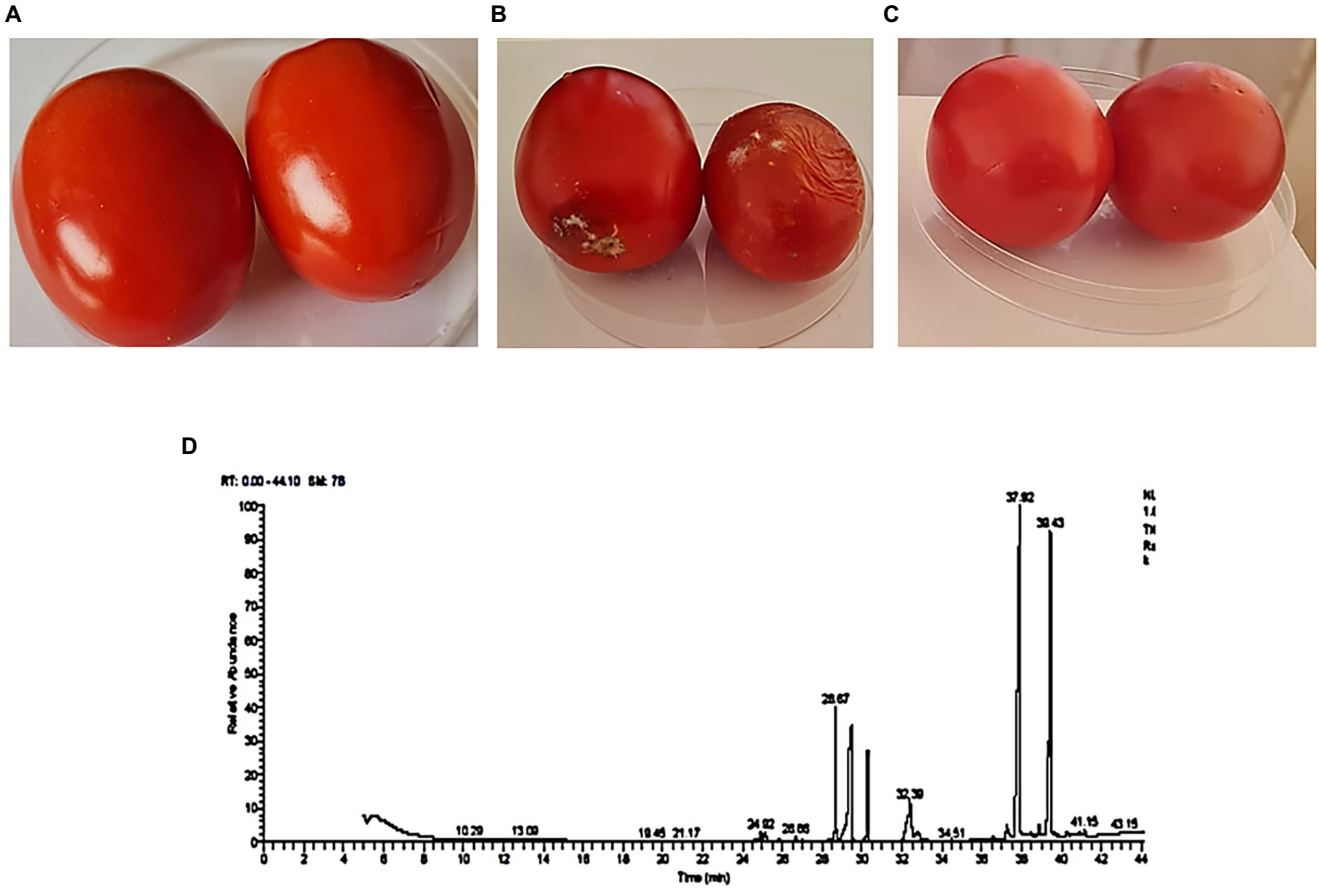
Biocontrol efficacy of *T. asperellum* crude extract *against F. oxysprum*. **(A)** Tomato fruit inoculated with *T. asperellum* extract. **(B)** Tomato fruit inoculated with a spore suspension of *F. oxysporum only*. **(C)** Tomato fruit inoculated with *T. asperellum* extract and *F. oxysporum*. **(D)** The fraction of bioactive secondary metabolites were detected using *a GC–MS* chromatogram obtained from *T. asperellum.*

The disease control efficacy of *T. asperellum* against *F. oxysporum* is shown in Figures 8A–C. After 11 days of storage, tomato fruits inoculated with *F. oxysporum* and treated with *T. asperellum* extract showed an increase in shelf-life compared with the untreated control (*Fusarium* only). Moreover, tomato fruits inoculated with *Trichoderma* metabolites showed a significant reduction in lesion diameter compared to the pathogen-treated fruits (Figure 8). Results indicated that *T. asperellum* exhibited a significant inhibitory effect against *F. oxysporum*. In addition, the mycotoxins typically produced by *Fusarium* species (FB1, FB2, ZEN, and DON) were not detected by LC–MS/MS analysis, even in the untreated controls.

### GC–MS analysis

The GC–MS analysis of the ethyl acetate extract of *T. asperellum* revealed the presence of different bioactive compounds (Figure 8D). The compound name, molecular formula (MF), molecular weight (MW), concentration (peak area %), and retention time (RT) are present in Table 3. Interestingly, the most predominant compounds were 1,2-Benzenedicarboxylic acids (32.15%), Palmitic Acid, TMS derivative (17.05%), D-Glucopyranose, 5TMS derivative(7.04%), Myo-Inositol, 6TMS derivative(4.55%), Oleic Acid, (Z)-, TMS derivative (2.82%), 9,12,15-OCTADECATRIENOIC ACID, 2-[(TRIMETHYLSILYL) OXY]-1-[[(TRIMETHYLSILYL) OXY] MET HYL]ETHYL ESTER, (Z, Z, Z)-(1.35%), 4H-1-Benzopyran-4-one,2-(3,4-Dihydroxyohenyl)-6,8-DI-á-D-Glucopyranosyl-5, 7-Dihydroxy (1.35%) and Stearic acid, TMS derivative (0.82%).

**Table 3:** Bioactive compounds identified in *T. asperellum* crude extract using GC–MS analysis.

T No.	R.T.	Area %	Name of the compound	Formula	Molecular weight
1	5.46	1.03	Methanol, TBDMS derivative	C7H18OSi	146
2	24.92	0.51	D-(−)-Fructofuranose, pentakis(trimethylsilyl) ether (isomer 2)	C21H52O6Si5	540
3	25.18	0.31	D-(−)-Ribofuranose, tetrakis(trimethylsilyl) ether (isomer 1)	C17H42O5Si4	438
4	26.66	0.23	D-(+)-Galactopyranose, 5TMS derivative (isomer 2)	C21H52O6Si5	540
5	28.67	7.04	D-Glucopyranose, 5TMS derivative	C21H52O6Si5	540
6	29.46	17.05	Palmitic Acid, TMS derivative	C19H40O2Si	328
7	30.28	4.55	Myo-Inositol, 6TMS derivative	C24H60O6Si6	612
8	32.40	2.82	9-Octadecenoic acid, (E)-, TMS derivative	C21H42O2Si	354
9	32.40	2.82	11-cis-octadecenoic acid 1TMS	C21H42O2Si	354
10	32.40	2.82	13-Octadecenoic acid, (E)-, TMS derivative	C21H42O2Si	354
11	32.40	2.82	Oleic Acid, (Z)-, TMS derivative	C21H42O2Si	354
12	32.83	0.82	Octadecanoic acid, trimethylsilyl ester	C21H44O2Si	356
13	32.83	0.82	10,12-Docosadiynedioic acid, 2TMS derivative	C28H50O4Si2	506
14	32.83	0.82	Stearic acid, TMS derivative	C21H44O2Si	356
15	32.83	0.82	5,8,11-Eicosatriynoic acid, tert-butyldimethylsilyl ester	C26H42O2Si	414
16	37.28	1.35	4H-1-Benzopyran-4-one,2-(3,4-Dihydroxyohenyl)-6,8-DI-á-D-Glucopyranosyl-5, 7-Dihydroxy	C27H30O16	610
17	37.28	1.35	9,12,15-Octadecatrienoic acid, 2-[(trimethylsilyl)oxy]-1- {[(trimethylsilyl)oxy] methyl}ethyl ester, (Z,Z,Z)-	C27H52O4Si2	496
18	37.91	32.15	1,2-Benzenedicarboxylic acid	C24H38O4	390

R.T, Retention time.

## Discussion

Various antagonistic activities were studied against different fungal pathogens using *T. asperellum,* as shown in Figure 3. This was sufficient evidence to conclude that the *Trichoderma* strain could be a bio-control agent and stimulate plant health *via* secondary metabolites and hormone production. In addition, this strain has the capability to sustain beneficial interaction with the host plant under stressful biotic conditions. Several *Trichoderma* isolates have been reported to suppress FWD (Srivastava et al., 2010; Marzano et al., 2013). *Trichoderma* species also have the potential of antagonistic interactions with different plant pathogens, which determines the biocontrol efficiency of the fungal pathogens. Mycoparasitism, antibiosis, competition for nutrients, and induced systemic resistance in plants are diverse antifungal activities of *Trichoderma* spp. (Druzhinina et al., 2011; Modrzewska et al., 2022). Therefore, these studies showed the successful application of *Trichoderma* in the field, which could impair plant health and productivity and suppress the *Fusarium* wilt disease. Additionally, this study investigated the contribution of *T. asperellum* to P solubilization and IAA production. Experiments were conducted in duplicate to investigate the ability of *Trichoderma* to promote plant growth. *Trichoderma* produced a clear halo around its colonies, which solubilized soluble P *in vitro*. Interestingly, *T. asperellum* released an auxin-like phytohormone that significantly increased the total root length of tomato plants. Since the tomato is one of the most produced crop plants growing in fields and greenhouses worldwide, this study evaluated the mechanism by which *Trichoderma* assisted tomato roots in response to *Fusarium* infection during plant growth. Plant roots exploit morphological plasticity to adapt and respond to different soil environments (Alaguero-Cordovilla et al., 2018). However, most of the investigated *Trichoderma* species colonize the root surface or inhabit inside root tissues as endophytes (Bailey et al., 2006). *Trichoderma* application with tomato seedlings shows adequate protection against *F. oxysporum*. Interestingly, the *T. asperellum* also benefitted plant growth promotion *via* root elongation during the pathogen attacks (Figure 5G). This is consistent with the dual cultural assay data between the biological agents and the pathogen infection, which decreased the hyphal growth. The results showed that the highest growth rate inhibition was displayed against *F. oxysporum*, causing FWD.

A precise *in vitro* experimental design was established to explore the impact of *Trichoderma* inoculation on the tomato root system during early growth (Figure 5D). Our results revealed that the biocontrol agent led to observed differences during early growth. The treatments were categorized into tomato roots with no microbial inoculation (control) and root inoculation with *Trichoderma*. The effect of *Trichoderma* strain on plant growth under abiotic stress was initially evaluated by measuring the root and shoot length and the number of leaves. In addition, the fungal inoculation significantly increased the plant parameters compared to untreated plants (control), as shown in Figure 5H. In that context, some studies have revealed that the inoculation of *T. harzianum* SQR-T037 improved the growth of tomato plants under greenhouse and field conditions (Cai et al., 2013, 2015).

The root system is essential for plant growth because of its basic functions in the selective absorption of water and nutrients (Karthika et al., 2018; Das et al., 2022), storage organ, and a selective barrier against pathogens (Wiedemeier et al., 2002; Atkinson et al., 2014). Characterization of root morphology and cellular development of two tomato species, *S. pennellii* and *S. lycopersicum* ‘M82’ during early growth were studied by Ron et al. (2013). This study provided significant differences in an extensive range of root traits with developmental significance. In addition, the newly emerged lateral roots (LRs) were significantly correlated with *Trichoderma* inoculation in tomato plants (Figure 7F). In addition, *Trichoderma* (green) colonized tomato roots (red), which visualized its mobility by the confocal imaging microscope (Figure 5J). It is well known that *Trichoderma* strains are able to colonize plant roots and stimulate the growth *via* the production of phytohormones, thus playing a crucial role in regulating the plant root system (Benítez et al., 2004; Harman et al., 2004; Cai et al., 2013). These findings agreed with our observations which suggested that improving plant growth occurs through a direct effect of *Trichoderma* species on root development.

Furthermore, *Trichoderma* can regulate plant growth through other mechanisms, such as mineral solubilization, to sustain plant health. These findings were consistent with the results found in this study when inoculating tomato seeds with *T. asperellum* in healthy conditions or under pathogenic stress. Tomato root colonization and *Trichoderma* mobility were visualized using confocal laser imaging (Figure 5J) in the rhizosphere to discover the tomato root-*Trichoderma* interactions. Tomato seeds were sterilized, germinated, and inoculated, and seedlings were grown under the conditions described (Simons et al., 1996). *Trichoderma* and *Fusarium* were labeled with autofluorescent protein (AFP) markers to visualize their interactions using confocal laser scanning microscopy (CLSM). This study better understood the biocontrol interaction and mycoparasitic activity (Bloemberg, 2007).

Similarly, our study showed the fungal mobility in the tomato rhizosphere, which stimulates root growth promotion (Bolwerk and Lugtenberg, 2005), as shown in Figures 5I,J, which illustrates the early and robust stage of tomato root colonization. In addition to direct parasitism of plant pathogens, interactions with *Trichoderma* enhance plant fitness in response to biotic and abiotic stresses (Bolwerk et al., 2005; Hermosa et al., 2012). All microbes generally hunt for food, of which the root supplies a substantial amount of exudate. Visualization of plant-microbe and microbe-microbe interactions during the plant developmental stages is essential to understanding the underlying mechanisms behind the root exudates/signaling and microbial colonization *via* tracking microbial mobility. In addition, it explores the various mechanisms of biological control by increasing tolerance to abiotic stress and stimulating defense strategies against fungal pathogens (Hermosa et al., 2012). Tomato seedlings inoculated with *Burkholderia tropica* strain MTo-293 exhibited some activities in plant growth promotion and biological control under greenhouse conditions (Bernabeu et al., 2015). They studied the performance of the microbial colonization of different vegetal tissues. A later confocal microscopy study also revealed that *T. harzianum* could colonize the epidermis and cortex of tomato roots (Chacón et al., 2007). In a recent study, *T. harzianum* T-78 enhanced the resistance of tomato plants against the root-knot nematode Meloidogyne incognita through priming for salicylic acid (SA)-and jasmonic acid (JA)-regulated defenses (Martínez-Medina et al., 2017). Root colonization by *Trichoderma* requires a complex molecular dialog between the fungus and plant. This colonization is limited to the outermost layers of the root and does not penetrate the plant vascular bundle, which increases the resilience of abiotic stresses (i.e., salinity and drought). In addition, improving the capacity to absorb nutrients and actively stimulates plant growth (Contreras-Cornejo et al., 2021). *T. atroviride* AN35 and *T. cremeum* AN392 colonized the roots of wheat plants. The microscopic imaging showed that the hyphae of the *Trichoderma* grew on the root surface of wheat and corn (*Zea mays*) seedlings (Contreras-Cornejo et al., 2018). This behavior was also observed in *T. atroviride* during the colonization of *Arabidopsis* roots (Salas-Marina et al., 2011). A study by Ruano-Rosa et al. (2016) showed the root colonization process between *T. harzianum* and olive crops.

*Trichoderma* species are known to produce a wide range of bioactive secondary metabolites that are known to have antifungal, antibacterial, and toxic properties to control a wide range of phytopathogens, such as *Fusarium* species, *Botrytis cinerea*, *Pythium* species, *Rhizoctonia solani*, *Sclerotinia sclerotiorum*, and *Ustilago maydis* (Hermosa et al., 2014; Khan et al., 2020). Analysis by GC –MS is essential for the identification of natural compounds of the microbe to explore the underlying mechanisms of the antifungal activity (Siddiquee et al., 2012; Qualhato et al., 2013; Meena et al., 2017). Usually, volatile compounds are identified, such as aromatic compounds, fatty acids, general hydrocarbons, and hydroxy or amino compound metabolites (Siddiquee et al., 2012). In the present study, 18 bioactive compounds were detected in the crude extract of *T. asperellum* by GC–MS analysis. The most commonly identified compounds were fatty acids and their derivatives, esters such as 1,2-Benzenedicarboxylic acid, Palmitic acids, Oleic acid, 9-Octadecenoic acid (E), methyl ester, and 9,12,15-Octadecatrienoic acid, methyl ester, (Z, Z, Z), sugars (D-Glucopyranose) and sugar alcohol (Myo-Inositol). It was investigated that sugars are essential to fuel the energy required for defenses and serve as signals for regulating defense genes in plant-microbe interactions (Roitsch et al., 2003; Bolton, 2009). Myo-inositol plays essential roles in stress responses, development, and many other processes (Michell, 2008; Valluru and Van den Ende, 2011). Moreover, the volatile metabolites play essential roles in mycoparasitic interactions between *Trichoderma* and plants (Altieri et al., 2009) 1,2 Benzenedicarboxylic acid has antifungal activity (Chen et al., 2020). Liu et al. (2008) emphasized the antifungal activity of palmitic acid against four economically important phytopathogenic fungi (*Alternaria solani*, *Colletotrichum lagenarium*, *Fusarium oxysporum* f. sp. *cucumerinum*, and *F. oxysporum* f. sp. *lycopersici*). Walters et al. (2004) examined the antifungal ability of oleic acid against plant pathogenic fungi. They verified that a 1,000 μM could reduce the mycelial growth of all tested fungi (*Rhizoctonia solani*, *Pythium ultimum*, *Pyrenophora avenae*, and *C. perniciosa*). The primary mechanism of the antifungal action of fatty acids states that fatty acids insert themselves into the lipid bilayers of fungal membranes compromising membrane integrity, resulting in an uncontrolled release of intracellular electrolytes and proteins, eventually leading to cytoplasmatic disintegration of fungal cells (Avis and Bélanger, 2001). Thus, these fatty acids play a vital role in the extract of *T. asperellum* by controlling the growth of *F. oxysporum*. Therefore, it protects tomato plants from *Fusarium* diseases.

Surprisingly, the mycotoxins naturally produced by *Fusarium* species (FB1, FB2, ZEN, and DON) were not detected by LC–MS analysis, even in the untreated controls. Our results agree with Stracquadanio et al. (2021), who showed that *F. graminearum* had no mycotoxin production in tomato fruits. Likewise, in the studies by Lori et al. (2003) and Haidukowski et al. (2005) on the same strain, *F. graminearum* ITEM 126 showed mycotoxin production in contaminated wheat kernels. This could be because this strain does not produce mycotoxins when infecting tomatoes but only in small grain cereals and maize (Munkvold, 2017).

## Conclusion

Tomato is a global economically essential vegetable crop. However, FOL is the causal agent of the FWD of tomato crops. Biological control offers a promising eco-friendly method to manage this disease. The *Trichoderma* strain is successfully used as a bio-control agent because it stimulates the plant immune system against pathogen attacks. Its growth-promoting ability in soil provides an additional benefit in the agricultural application of fertilizers and antifungal activity. Furthermore, a combination of the *in vitro* and *planta* experiments will improve the role of this fungus on plant performance. In addition, the fungus successfully promotes plant growth in controlled conditions. The plant growth-promoting traits and biocontrol efficiency of the *Trichoderma* strain were also evaluated based on the recurring role of *T. asperllum* as a biocontrol agent against various fungal pathogens. *T. asperellum* displayed the highest antagonistic activity against *F. oxysprum* at 53.24%, and 30% free cell filtrate inhibited *F. oxysporum* at 59.39% as well. This study provides vital insights into the plant-microbe interaction and the microbe-microbe interactions, including diverse antagonistic mechanisms. For instance, chitinase activity, IAA, and P solubility were observed *in vitro* assays, indicating *Trichoderma’s* capability to control the fungal pathogen and enhance plant growth. Additionally, using confocal microscopy, tomato root colonization was visualized to understand the mobility of the *Trichoderma* strain in the host plant. These data suggest microbial application in seed coating or foliar spraying, which may improve food safety by applying beneficial microbes. In sum, improving the efficacy and development of biocontrol agents to help small farmers will increase their crop productivity in an accessible and economical way. Thereby positively impacting farmers’ profits and sustaining the food safety approaches. In addition, developing sustainable crops that could be grown with little to no pesticides and/or chemical fertilizers reduces costs for farmers in developing areas. Moreover, developing novel and environmentally friendly approaches is essential to reduce the FWD incidence and yield loss in tomato crops. Therefore, more field applications with this *Trichoderma* strain are required. In addition, the transcriptome profiles of the root system following microbial treatment and the influence of endophytic fungi on tomato metabolism.

## Data availability statement

The datasets presented in this study can be found in online repositories. The names of the repository/repositories and accession number(s) can be found in the article/Supplementary material.

## Author contributions

AS, RA, KA, and OH designed the study. AS, RA, and OH wrote the original manuscript. AS and RA conducted all the in vitro experiments, confocal work, and collected the data. AS, RA, and OH conducted the in planta assays. OH performed the ITS sequencing to confirm fungal genus taxonomy. OH, KA, and MA performed the statistical analysis and edited the manuscript. All authors contributed to the article and approved the submitted version.

## Conflict of interest

The authors declare that the research was conducted in the absence of any commercial or financial relationships that could be construed as a potential conflict of interest.

## Publisher’s note

All claims expressed in this article are solely those of the authors and do not necessarily represent those of their affiliated organizations, or those of the publisher, the editors and the reviewers. Any product that may be evaluated in this article, or claim that may be made by its manufacturer, is not guaranteed or endorsed by the publisher.

## Footnotes

^1^http:%20www.ncbi.nlm.gov/BLAST/
