# Supplementary material for: Trichoderma asperellum empowers tomato plants and suppresses Fusarium oxysporum through priming responses
Source: Front Microbiol. 2023 Mar 14;14:1140378. doi: 10.3389/fmicb.2023.1140378 (PMC10043483; doi:10.3389/fmicb.2023.1140378)
Supplement: Supplementary file 1 [file Data_Sheet_1.docx]

**Fig. S1**. Effect of *T. asperellum* treatment on tomato seed germination and Vigor index.
